# Supplementary material for: Genome-scale metabolic reconstruction of the symbiosis between a leguminous plant and a nitrogen-fixing bacterium
Source: Nat Commun. 2020 May 22;11:2574. doi: 10.1038/s41467-020-16484-2 (PMC7244743; doi:10.1038/s41467-020-16484-2)
Supplement: Supplementary file 1 — Supplementary Information [file 41467_2020_16484_MOESM1_ESM.pdf]

Supplementary Information

Genome-scale metabolic reconstruction of the symbiosis between a leguminous plant and a nitrogen-fixing bacterium

George C diCenzo[1,2], Michelangelo Tesi[1], Thomas Pfau[3], Alessio Mengoni[1*], and Marco Fondi[1*]

[1] Department of Biology, University of Florence, Sesto Fiorentino, Italy

[2] Department of Biology, Queen's University, Kingston, Ontario, Canada

[3] Life Sciences Research Unit, University of Luxembourg, Belvaux, Luxembourg

* **Corresponding authors**: Alessio Mengoni (alessio.mengoni@unifi.it) and Marco Fondi (marco.fondi@unifi.it)

Supplementary Note 1. Preparing an improved *S. meliloti* metabolic reconstruction.

A new *S. meliloti* metabolic reconstruction was built using the existing core metabolic reconstruction iGD726 [1] as a starting point. First, the biomass composition was updated as summarized in Supplementary Data 7 ("Biomass reaction"). In particular, glycogen was reduced to 0.1% cell dry weight (CDW), poly-hydroxybutyrate was reduced to 1% CDW, and high and low molecular weight succinoglycan were reduced to 0.1% and 0.4% CDW, respectively [2]. Additionally, putrescine and spermidine were added to the biomass composition at trace concentrations [3].

The working reconstruction was manually expanded to contain accessory metabolic pathways following our previously reported workflow [1]. Briefly, the reconstruction was expanded by the addition of one pathway at a time. For each pathway, reactions were individually added to the model, the gene associations and reaction equations checked against literature sources, and where possible, each reaction was referenced (see Supplementary Materials S2). Reactions were predominately taken from the previously published *S. meliloti* genome-scale metabolic reconstruction iGD1575 [4] when possible; otherwise, they were taken from the Kyoto Encyclopedia of Genes and Genomes [5], MetaCyc [6], ModelSEED [7], or MetaNetX [8] databases.

An automated expansion of the metabolic network was then performed. Using the 'tncore_expand' function of the Tn-Core Toolbox, all reactions absent in the working reconstruction but present in the *S. meliloti* genome-scale metabolic reconstruction iGD1575b [1] were transferred to the working reconstruction. Then, i) all unnecessary 'source' reactions were removed, ii) most metabolic reactions associated with an unknown gene were removed, and iii) some newly added reactions likely to be incorrect based on published literature were deleted. Reactions added during the automated expansion sharing a gene in common with an existing reaction were then manually examined, and in most cases manually removed from the reconstruction. Then, all reactions producing dead-end metabolites were iteratively removed,

The working reconstruction was next mass and charge balanced. Metabolite formulas and charges were obtained from the MetaNetX database [8] when available; otherwise, metabolite charges and formulas were manually prepared, using information from the PubChem database [9] when available. The 'checkMassChargeBalance' function of the COBRA Toolbox was used to identify mass or charge unbalanced reactions, and reaction equations were manually balanced. Duplicate reactions were identified and removed.

An ATP hydrolysis reaction was added to account for non-growth associated maintenance (NGAM) costs [10], using a NGAM cost of 8.39 mmol ATP h^{-1} (g dry weight)$^{-1}$ as reported for *Escherichia coli* [11]. A growth associated maintenance (GAM) reaction was not added as the reconstruction includes transcription and translation reactions. The final reconstruction, termed iGD1348, contains 1348 genes, 1407 reactions (1164 associated with at least one gene), and 1160 metabolites (Table S1). The final reconstruction is available in Supplementary Software in SBML, XLS, and MATLAB COBRA format.

Supplementary Note 2. Updating the *M. truncatula* metabolic network reconstruction.

The published *M. truncatula* metabolic network reconstruction [12] was built based on the *M.*

truncatula genome version Mt3.5v5 [13]. Here, the gene associations were updated to correspond to the annotations of version 5.0, the most recent version of the *M. truncatula* genome [14]. A conversion table was prepared linking the Mt3.5v5 gene names with the corresponding gene names from the Mt4.0v1 genome annotation [15], which were in turn associated with the corresponding gene names from the version 5.0 annotation. This conversion table was prepared based on the information present in i) the 'Mt3.5-Mt4.0v1_conversion_table.txt' file available on medicagogenome.org [16], and ii) the 'MtrunA17r5.0-ANR-EGN-r1.6.gene-repeat_region.vs. JCVI-Mt4.0-gene.kgb.synonymy.txt' file available online at medicago.toulouse.inra.fr/ MtrunA17r5.0-ANR. Next, the published *M. truncatula* SBML model was imported into MATLAB with the 'importMedicago' function of Pfau *et al.* [12]. After importing, for genes with a one-to-one match between genome versions, the existing gene name was replaced with the gene name in the version 5.0 genome annotation. When multiple genes were combined into a single gene in the version 5.0 annotation, all of the genes were removed from the model and replaced with the single gene. Genes that were split into multiple genes in the version 5.0 annotation were replaced with all of the new genes using an 'or' association. Genes with no match in the Mt5.0 genome were removed from the model; reactions constrained upon removing these genes were also deleted unless they were essential for model growth (i.e., RXN-9944_H, RXN-7674_H, and PASTOQUINOL--PLASTOCYANIN-REDUCTASE-RXN_H), in which case the corresponding gene also was not removed.

The majority of transport reactions in the original *M. truncatula* metabolic reconstruction, both between the cell and the external environment and between organelles, were simple diffusion reactions lacking an energy source such as ATP hydrolysis or proton cotransport. To limit inappropriate transport between compartments, all single-metabolite diffusion reactions were modified with the exception of metabolites such as water, gases, and light. All bidirectional reactions were split into two unidirectional reactions, and each reaction was modified to require the hydrolysis of 0.25 mol of ATP per mol of transported compound. The modified reconstruction contains 2522 genes, 2920 reactions (1722 associated with at least one gene), and 2742 metabolites.

The updated *M. truncatula* reconstruction was used to generate a tissue-specific *M. truncatula* model containing shoot and root tissues using the 'BuildTissueModel' function of Pfau *et al.* [12]. Reactions to transfer metabolites between the root and shoot tissue were modified to require the hydrolysis of 0.25 mol of root ATP and 0.25 mol of shoot ATP per mol of transferred metabolite. The model was then modified to contain unique gene names for those associated with the shoot tissue and for those associated with the root tissue, following which all unused genes were removed from the model. Finally, root import reactions for the following compounds were added in anticipation of integration with the *S. meliloti* model: Co^{2+}, MoO_4^{3-}, Mn^{2+}, Zn^{2+}, Ca^{2+}, K^+, and Na^+. The final model encompassed root metabolism and shoot metabolism with appropriate cross-talk between the tissues [12], and all reactions, metabolites, and genes associated with the shoot contain the prefix 'Leave_', while those associated with the root contain the prefix 'Root_'. The final reconstruction is available in Supplementary Software in SBML, format.

Supplementary Note 3. Adding sucrose metabolism to zone III bacteroids in ViNE.

To perform simulations comparing the use of sucrose and C_4-dicarboxylates as the carbon source for zone III bacteroids, ViNE was modified as follows. The pipeline for construction of ViNE, as detailed below in Supplementary Note 4, was rerun with a single change. Prior to running GIMME, reactions for the import of all carbon sources, except sucrose, into the symbiosomes were deleted. This is in contrast to the construction of the regular version of ViNE, when the reactions for the import of all carbon sources, except C_4-dicarboxlates, into the symbiosomes were deleted. The resulting model was then combined with ViNE, producing an enlarged version of ViNE supplemented with the necessary reactions to allow sucrose to serve as a carbon source for zone III bacteroids.

Supplementary Note 4. Reconstructing the metabolism of a nodulated *M. truncatula* plant.

The original full (i.e., non-tissue-specific) *M. truncatula* reconstruction [12] was imported to MATLAB in COBRA format from SBML format using the 'readCbModel' function. The model was updated to the version 5.0 genome annotations as described in the previously section, and diffusion reactions were modified to require an energy source as described in the previous section. The following reactions were then added in preparation for integration with the *S. meliloti* model: a homocitrate synthase reaction, a biotin source reaction, a H_2 export reaction, and import reactions for each of N_2, Mn^{2+}, Zn^{2+}, Ca^{2+}, K^+, and Na^+. The gene *MtrunA17Chr1g0213481* was associated with the homocitrate synthase reaction based on homology to the gene of *Lotus japonicus* [17]. At the same time, the *S. meliloti* model was modified such that fluxes were recorded in μmol hr^{-1} (g dry weight)$^{-1}$, with one μmol of biomass equalling one g of biomass. This was done to ensure consistency with the units in the *M. truncatula* model. The *S. meliloti* model contained a single gene for all unknown GPRs (i.e., 'Unknown') and a single gene for all spontaneous reactions (i.e., 'Spontaneous'). In preparation for constraining the nodule, the 'Unknown' and 'Spontaneous' genes were replaced with a series of genes each associated with a single reaction.

The following strategy was adopted to build a multi-compartment metabolic model accounting for the metabolic interactions of the two organisms. First, we mapped the two reconstructions to the same name space using the MetaNetX version 3.1 source files [8]. This step was necessary as the *S. meliloti* model is based on the SEED database [18] and the *M. truncatula* model on the MetaCyc database [6]. To minimize the required adjustments, only the metabolite identifiers of metabolites that were both i) a boundary metabolite in the *S. meliloti* model and ii) a cytoplasmic compound in the *M. truncatula* model were changed to the corresponding MetaNetX code; these represent the pool of metabolites that can be exchanged between the organisms. A multi-compartment reconstruction was built that included the *M. truncatula* model, the *S. meliloti* model, and transport reactions (without gene associations) that convert the *M. truncatula* cytoplasmic compounds to extra-cellular *S. meliloti* compounds (e.g., H2O_C => cpd00001[e]). These reactions represent the transport of compounds across the peribacteroid membrane, between the *M. truncatula* cytoplasm and the peribacteroid space of the symbiosome. For each metabolite, two unidirectional transport reactions were added that each required the hydrolysis of 0.25 mol of plant ATP per mol of the metabolite of interest. The exception was ammonia; in this case, the

transport reaction into the peribacteroid space was driven by the hydrolysis of 0.25 mol of ATP per mol of ammonia, while the transport from the peribacteroid space was driven by proton symport (one proton per one molecule of ammonia). Additionally, protons transferred to the peribacteroid space from the *M. truncatula* cytoplasm were separated from protons exported by *S. meliloti*; no exchange of protons between *S. meliloti* and *M. truncatula* was allowed at this stage.

Four copies of the integrated *M. truncatula – S. meliloti* model were prepared to represent four distinct developmental zones of the nodule: zone II distal, zone II proximal, interzone II-III, and the nitrogen-fixing zone III. Additionally, a version of the *M. truncatula* model prior to integration with *S. meliloti* was included to represent zone I (apical meristem). In each of the five models, prefixes were added to all reactions, metabolites, and genes to specify to which zone and which organism the feature belongs (e.g., 'NoduleIII_' and 'BacteroidIII_'). Next, all *S. meliloti* exchange reactions and all *M. truncatula* transport reactions were deleted in each of the five models. The exception was for nodule zone III, where the import of N_2 and export of H_2 by *M. truncatula* were not removed. Finally, a single model was produced that joined the tissue-specific (root and shoot) *M. truncatula* model with the five nodule zone models as a single COBRA formatted metabolic model. To this model, an irreversible reaction converting protons in the peribacteroid space to *S. meliloti* periplasmic protons was added specifically in nodule zone III, thereby allowing the transfer of protons from *M. truncatula* to *S. meliloti*.

At this point, it was necessary to metabolically connect the nodule to the root and to the external environment. First, for each compound that could be exported by the *M. truncatula* root tissue, a reaction was added to each of the five nodule zones for the export of that compound. Then, for all compounds that could be imported by the *M. truncatula* root tissue (except ammonium and nitrate), a diffusion reaction (without an energy requirement) was added for the import of the metabolite from the external environment to a general nodule compound. Next, all compounds were identified that could be transferred between the root and shoot tissues in either direction. For each of these compounds, a diffusion reaction (without an energy requirement) was added to convert the compound in the root to a general nodule compound. Then, for each of the general nodule compounds, five irreversible reactions were added to transfer the general nodule metabolite to each of the nodule zones; each reaction involved the hydrolysis of 0.25 mol of nodule zone ATP per mol of transported metabolite. Finally, reactions were added to individually transfer asparagine and glutamine from the *M. truncatula* plant cytoplasm of nodule zone III (the nitrogen-fixing zone) to the root tissue, with each reaction requiring the hydrolysis of 0.25 mol of root ATP and 0.25 mol of nodule zone III ATP per mol of metabolite.

A series of biomass reactions were added to the combined model. A zone-specific biomass reaction was added to each of zone II distal, zone II proximal, and interzone II-III by combining *M. truncatula* and *S. meliloti* biomasses at a 75 : 25 ratio. Biomass of zone I consisted of only *M. truncatula* biomass. No biomass reaction was added to zone III as the purpose of this zone was to fix nitrogen. Next, an overall nodule biomass reaction was prepared by combining zone I, zone II distal, zone II proximal, and interzone IZ biomass at a 5 : 45 : 45 : 5 ratio. A plant biomass reaction was also prepared by combining shoot and root biomass at a 66.7 : 33.3 ratio. Finally, an overall

biomass reaction was prepared that combined plant biomass with nodule biomass at a 98 : 2 ratio. The overall biomass reaction was set as the objective function during all FBA simulations unless stated otherwise.

All reactions that produced dead-end metabolites were iteratively removed from the model, followed by the addition of several constraints into the model (the list of the reactions removed following this procedure are listed in Supplementary Data 9). Maintenance costs, in the form of ATP hydrolysis, were added to each tissue including the nitrogen-fixing zone III. The maintenance cost value for the shoot and root tissues were set as described elsewhere [12]. Maintenance costs for plant nodule tissues were based on the shoot plus root maintenance costs scaled by the percent of biomass that consisted of the given nodule zone. Similarly, the maintenance costs of the bacteroid nodule tissues were based on 2.52 mmol hr^{-1} (g bacteria dry weight)$^{-1}$, scaled by the percent of total plant biomass that consisted of bacteroids of the given nodule zone; a value of 2.52 mmol hr^{-1} (g bacteria dry weight)$^{-1}$ was chosen as it equals 30% the commonly used value for free-living *Escherichia coli*. Import of ammonium and nitrate by the root and nodule tissues was turned off, as was usage of starch as a carbon source in the shoot tissue. The uptake of light was set to 1000 µmol hr^{-1} (g plant dry weight)$^{-1}$, which is within the range where there is a linear relation between light and CO_2 usage (not shown). The total rate of oxygen usage by the plant and bacterial cells of nodule zone III was limited to 8.985 µmol hr^{-1} (g plant dry weight)$^{-1}$. This value was arrived at as follows: i) the total oxygen usage of the entire nodule was limited to 12.98 µmol hr^{-1} (g plant dry weight)$^{-1}$ based on published experimental data [19], ii) plant growth was optimized, iii) the O_2 usage of zone III was limited to the O_2 uptake rate in the initial analysis, and iv) the constraint on whole nodule O_2 usage was removed. To force the use of C_4-dicarboxylates by the bacteroids of zone III, reactions for the import of all other carbon sources into the symbiosomes were deleted. No constraints were pre-set on the transfer of nutrients from the plant cytosol to the bacteria of zone II distal, zone II proximal, or interzone IZ. Finally, the upper and lower bounds of all reactions were multiplied by 1000, converting the units to nmol hr^{-1} (g dry weight)$^{-1}$. This step was necessary to avoid numerical issues when running GIMME due to low fluxes through the bacteroid reactions.

The reaction space of each nodule zone was constrained based on the *M. truncatula – S. meliloti* zone-specific RNA-seq data of Roux and coworkers [20], reanalyzed as described below, to obtain transcript per million (TPM) values. The expression threshold for a gene to be considered highly expressed was determined separately for each species, and it was equal to 1.1 times the average TPM value across all nodule zones of all genes that had at least one mapped read in at least one zone. To limit artificial differences between zones due to the choice to threshold, Kruskal-Wallis tests, followed by post-hoc comparisons, were performed for each gene to determine statistically significant between-zone expression changes; this was performed using the 'agricolae' package in R [21]. If i) the difference between two zones was not statistically significant, ii) only one of the two zone had an expression value above the expression threshold, and iii) the value in the second zone was at least 80% of the expression threshold, then the value of the second zone was modified to be above the expression threshold. Moreover, as we wished to only constrain the reaction space of the nodule zones, all shoot and root genes were given artificial values above the

expression threshold in order to ensure they were considered highly expressed.

The combined model was constrained using a custom multi-species adaptation of the gene-centric TIGER [22] implementation of the GIMME algorithm [23], which is available in the Tn-Core Toolbox [24]. In short, GIMME was modified to take multiple gene lists (one list per species), multiple TPM lists (one list per species), and multiple expression thresholds (one per species). Genes above the respective expression threshold were considered expressed, and those below the respective threshold were turned off. A score for each 'off' gene was calculated by subtracting the expression value of each gene from the appropriate threshold. The scores for the species were then normalized based on the ratio of the expression thresholds. The normalized values of both species were combined as a single list, and the GIMME algorithm continued as normal. The growth fraction threshold for GIMME was set to 0.99.

The GIMME output was used as the basis to build a constrained and functional COBRA-formatted model. As the genes identified as 'on' following the GIMME analysis were insufficient to rebuild a working COBRA model, the following pipeline was used. All reactions active during the GIMME analysis with an absolute flux > 1 x 10^{-6} nmol hr^{-1} (g dry weight)$^{-1}$ were identified. FASTCORE [25] (epsilon of 1.01 x 10^{-6}) was then run using these reactions as the input core reaction set and the same model used as input for GIMME, but with the lower bound of the biomass reaction set to 99% of the objective value. A list of protected reactions was prepared by combining: i) the output reactions of FASTCORE, ii) all reactions that were not constrained when the genes identified as 'off' in the GIMME analysis were deleted from the input model, iii) all peribacteroid transport reactions, and iv) all reactions for the transfer of metabolites between tissues. All nodule or bacteroid reactions that were not part of this protected list were removed from the model, and all genes no longer associated with a reaction were deleted. The genes associated with each reaction were then refined based on the GIMME output. For any given reaction, no change was made if all the associated genes were classified as 'on', or if all genes were linked with 'and' statements. Otherwise, for reactions with 'or' statements, but lacking 'and' statements, all genes classified as 'off' were removed from the reaction; if no gene was classified as 'on', then all genes were deleted except for the gene with the highest expression value. For reactions with both 'or' and 'and' statements, a complex loop was prepared. Put briefly, a minimal set of genes required for the reaction to be functional was left associated with the reaction, favouring the inclusion of 'on' genes followed by the inclusion of highly expressed 'off' genes. All reactions producing dead-end metabolites were iteratively removed from the model, and all genes no longer associated with a reaction were deleted. Finally, all reaction and metabolite identifiers were updated to MetaNetX codes, where possible, to maximize consistency throughout the model, and duplicate reactions were deleted. We refer to this final version of the integrated model as ViNE (for Virtual Nodule Environment), and it is provided in Supplementary Software as MATLAB COBRA and SBML formatted files.

Supplementary Note 5. Rationale for choice of between-tissue metabolite transfer reactions. ViNE contains many reactions moving metabolites between compartments, and the transporters that are included are likely to have an impact on the downstream simulations. Unfortunately, there

is not much information on the transfer of metabolites between these tissues in the literature. Thus, in deciding which transfer reactions to include in ViNE, we chose to start with a relatively unrestrictive approach, and then allow the integration of the RNA-seq expression data to guide the selection of which transfer reactions would be active by turning on/off metabolic pathways that would act on the transferred metabolites.

The metabolites allowed to move between the root and shoot tissues were taken from the previously published *M. truncatula* model [12], and so we assume them to be reasonable. The metabolites allowed to move between the root and nodule include the same metabolites that can transfer between the root and shoot (if these metabolites are moving between the root and shoot, they must be in the phloem and thus also able to move into the nodule tissue) and anything that can be imported by the root from the soil (as we assume the nodule tissue can transport the same compounds). As little is known about the transfer of metabolites between the plant cytosol and the bacteroids in the nodule, especially outside of zone III, we were unrestrictive; any compound in the cytosol for which *S. meliloti* has a transporter was allowed to be transported (at least prior to the integration of the RNA-seq expression data).

To test the impact of the inclusion or absence of individual reactions for the between-tissue transfer of metabolites, we tested the effect of individually deleting each of these reactions on the overall rate of plant growth in the unconstrained model prior to integration of RNA-seq data. Of the 110 reactions to move metabolites between the root, shoot, or nodule, the deletion of nearly half (53 reaction) had less than a 5% impact on overall plant growth. Of the 57 with an impact, all of them represent either key metabolites expected to be transferred (e.g., sucrose) or essential nutrients or micronutrients (e.g., sulfate, metal ions) that could only be imported from the external environment by only one tissue and are thus expected to be transferred to the other tissues. Of the 768 initial reactions for the transfer of metabolites between the plant and bacteria in the nodule, the deletion of only 53 (7%) had more than a 5% impact on overall plant growth. These 53 reactions transport essential micronutrients and minerals and gases like oxygen and nitrogen, and thus are expected to be required. Overall, these simulations support that the specific choice of between-tissue transfer reactions has little impact on the overall plant growth rate (the main output considered in this work), although we cannot rule out an effect on flux distribution.

Supplementary Note 6. Procedures for running Memote.

All the reconstructions were tested and evaluated using Memote [26]. The detailed reports are provided as Supplementary Software. In order to be able to run Memote on ViNE we had to skip a few tests of the default test pipeline, due to the size of this reconstruction compared to standard ones. In detail, we used the following command line for each of the three reconstructions tested herein:

```
docker run -v <path to model>:/opt opencobra/memote memote report
snapshot --filename "<path to out>report.html" /opt/model.xml --
skip  test_find_metabolites_not_consumed_with_open_bounds  --skip
test_find_metabolites_not_produced_with_open_bounds        --skip
```

```
test_find_metabolites_not_produced_with_open_bounds         --skip
test_find_metabolites_not_consumed_with_open_bounds         --skip
test_find_incorrect_thermodynamic_reversibility             --skip
test_biomass_open_production
```

Supplementary Note 7. Analysis of the RNA-sequencing data.
The nodule zones in the integrated metabolic model were constrained based on previously published RNA-seq data [20]; however, the data were first re-analyzed using the *M. truncatula* version 5 genome sequence and annotations. The raw sequencing reads (fastq format; SRA accession SRP028599) were downloaded from the European Nucleotide Archive database [27], and all files corresponding to the same replicate of the same zone were concatenated as a single file, keeping separate files for each mate pair. The *M. truncatula* A17 genome (version 5.0) and the *S. meliloti* Rm2011 genome were downloaded and combined as a single file. The combined genome was indexed using the bowtie2-build function with default settings [28]. Sequencing reads were mapped to the genome with bowtie2 version 2.2.3 [28], treating reads as paired-ends and using 20 threads. Output SAM files were sorted by name with samtools sort version 1.3.1-39-ga9054c7 [29] using 20 threads. Reads per gene were counted using HTseq-count version 0.6.1p1 [30], with the default alignment score threshold of 10. Each HTseq-count output table was split into two tables: one for *M. truncatula* and one for *S. meliloti*. TPM values were calculated for each species-specific output file using a custom Perl script, based on the total gene length for the *S. meliloti* genes and the total exon length for the *M. truncatula* genes. Finally, zone-specific average TPM values based the three biological replicates of each zone were calculated. These values were used to constrain the reaction space of the nodule zones in the integrated metabolic model.

Supplementary Note 8. Simulations involving varying rates of nodulation.
The following strategy was used for simulations where NH_4 was added to the soil and the rate of nodulation (defined as the rate of N_2-fixation per gram nodule) was optimized to maximize plant growth while the N_2-fixation efficiency (defined as the rate of N_2-fixation per gram nodule) remained constant (summarized in Supplementary Figure 4). First, the model was optimized in the absence of exogenous NH_4. Next, nodule biomass was removed from the overall biomass reaction and instead forced through a sink reaction at a rate equal to its synthesis in the previous step. The desired rate of NH_4 availability was then set and plant biomass production optimized. Based on the rates of plant growth and N_2-fixation in the previous step, and the desired N_2-fixation efficiency, the rate that nodule biomass was produced was updated. Plant biomass production was again optimized, and the rate of nodulation was used to update all nodule maintenance cost reactions. The following loop was then employed. Plant biomass production was optimized and the rates of plant growth and N_2-fixation, together with the desired N_2-fixation efficiency, were used to update the rate of nodulation. Plant biomass production was again optimized, and the rate of nodulation was used to update all nodule maintenance cost reactions. Once again, plant biomass production was optimized, and the maximal rate of zone III oxygen uptake was set based on the

rate of nodulation and the desired oxygen limit. Plant biomass production was optimized, and the N_2-fixation efficiency was calculated. This loop was iterated until the N_2-fixation efficiency was within 0.1% of the desired value, at which point the results were recorded.

The following strategy was used for simulations where the N_2-fixation efficiency (defined as the rate of N_2-fixation per gram nodule) was varied while the rate of nodulation (defined as the rate of N_2-fixation per gram nodule) was optimized to maximize plant growth (summarized in Supplementary Figure 5). First, the model was optimized with default settings, following which nodule biomass was removed from the overall biomass reaction and instead forced through a sink reaction at a rate equal to its synthesis in the initial FBA solution. The following loop was then employed. the maximal rate of zone III oxygen uptake and N_2-fixation was adjusted based on the rate of nodulation. The rate of plant biomass production was then optimized. Based on the rate of N_2-fixation in the solution of the previous step, the rate of nodulation was updated, the lower limits of all nodule maintenance cost reactions were updated, and the maximal rate of zone III oxygen uptake and N_2-fixation were updated. The rate of plant biomass production was again optimized. This loop was iterated until the N_2-fixation efficiency was within 0.5% of the desired value. We found that this loop only identified local maximums for the rate of plant biomass production. In order to find the global maximum, a second loop was employed. In each loop, the rate of nodulation was slightly increased relative to the previous solution. Next, the previous loop was again employed to identify the local maximum. This was repeated until the local maximum following a slight increase in the rate of nodulation was lower than the previous local maximum; we assume this meant the previous iteration represented the global maximum. The results for the global maximum were then recorded.

To examine the effect of N_2-fixation efficiency on plant growth in the absence of a zone III oxygen limitation, the same process was employed with the exception that the maximal rate of zone III oxygen uptake was kept constant at 1,000 μmol hr^{-1} (g plant dry weight)$^{-1}$. In cases where the rate of nodulation was capped at 5% of 10%, the same process was used with the exception that the rate of nodule biomass production was limited to 5% or 10% the sum of plant and nodule biomass production.

Supplementary Table 1. Summary properties of the *S. meliloti* metabolic reconstruction iGD1348.

**Genes**	1348
**Metabolites**	1160
Intra-cellular	989
Extra-cellular	171
**Reactions**	1407
Gene-associated reactions	1164
Metabolic reactions	1019
Gene-associated metabolic reactions	982
Transport reactions	197
Gene-associated transport reactions	179
Exchange reactions	169
Sink reactions	8
Source reactions	2
Biomass reactions	1
Other reactions	11

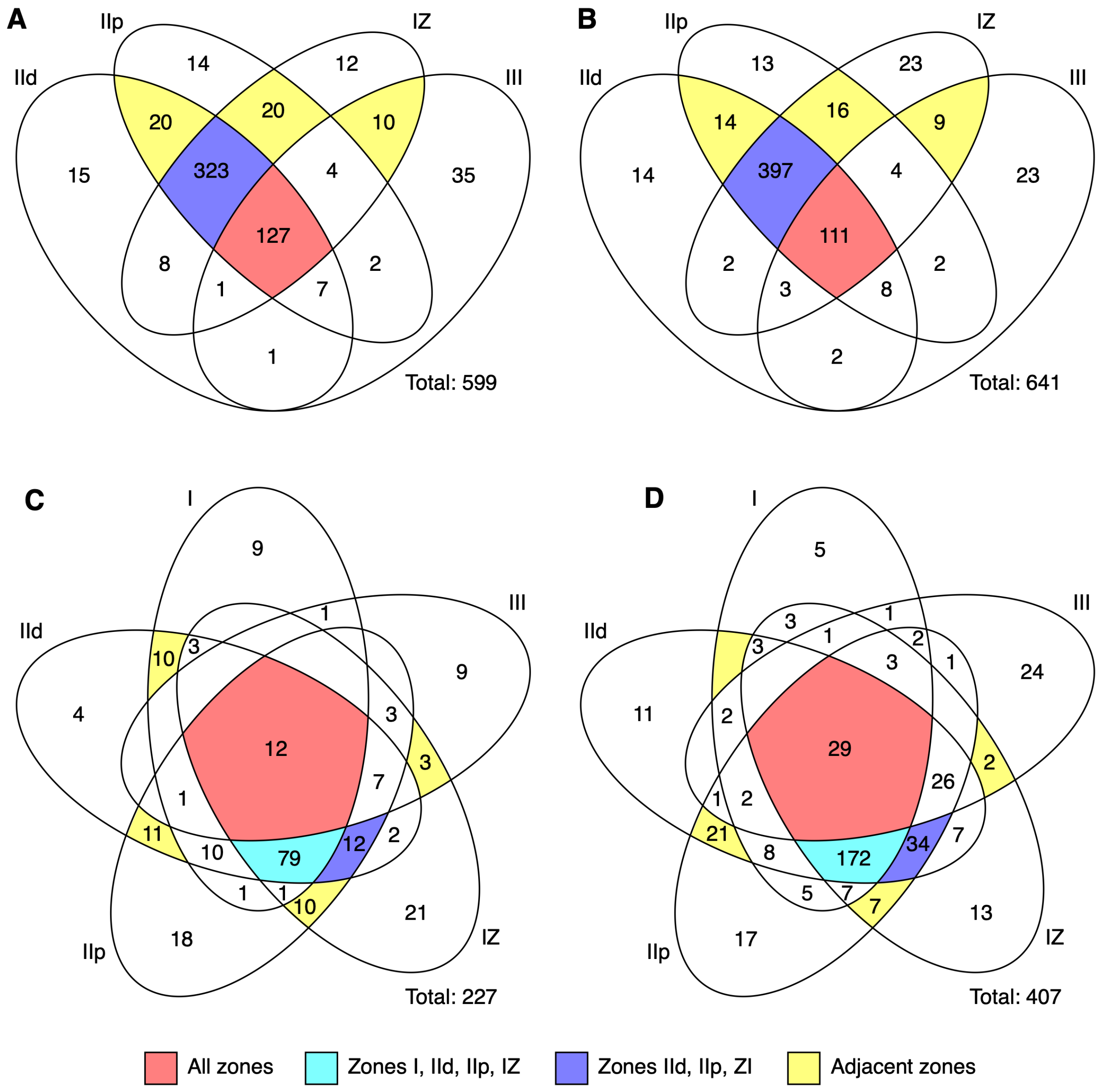

Supplementary Figure 1. Nodule zone-specific analysis of essential metabolism. Venn diagrams are presented showing the overlap in genes or reactions predicted to be essential with FBA (growth rate ratio < 0.1 compared to the wild-type model) in each nodule zone. The total number of essential genes or reactions in each Venn diagram is indicated. Venn diagrams are shown for (**A**) *S. meliloti* genes, (**B**) *S. meliloti* reactions, (**C**) *M. truncatula* genes, (**D**) *M. truncatula* reactions. Notable sections of the Venn diagram are coloured according to the legend provided in the figure. The absence of a number in a Venn diagram section indicates there were zero genes or reactions with that characteristic.

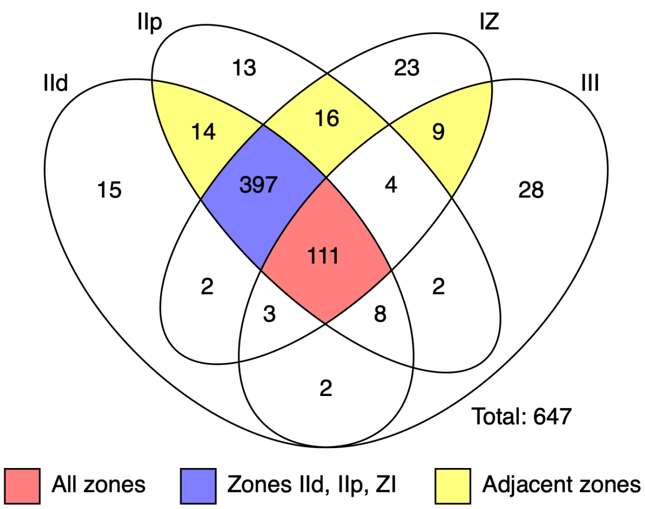

Supplementary Figure 2. Bacteroid robustness analysis summary. An analysis was performed to evaluate the effects of perturbing the flux through each bacteroid reaction, specifically in each nodule zone, on the predicted rate of plant growth. A Venn diagram is presented to summarize the overlap in the "synergistic" bacteroid reactions, which are the bacteroid reactions that had to carry non-zero flux (i.e., they had to be active) in order for the predicted rate of plant growth to be at least 95% the maximal predicted plant growth rate. Notably, there is extremely high overlap between the essential reaction set (i.e., reactions whose removal results in a predicted plant growth rate less that 10% the maximal predicted plant growth rate) and the synergistic reaction set. Notable sections of the Venn diagram are coloured according to the legend provided in the figure.

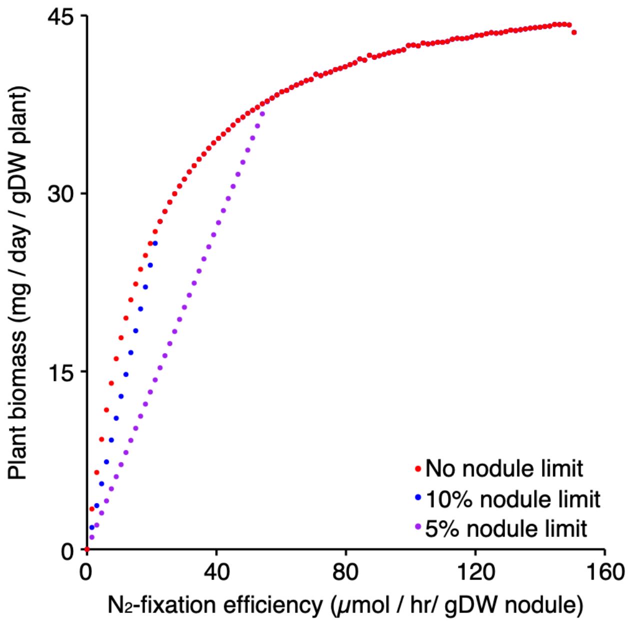

Supplementary Figure 3. Effect of N_2-fixation efficiency on plant biomass production. The effect of N_2-fixation efficiency (defined as the rate of N_2-fixation per gram nodule) on the rate of plant growth, with the rate of nodulation optimized to maximize plant growth. A constant upper limit on the rate of oxygen uptake by zone III nodule tissue (adjusted based on the ratio between nodule and plant biomass) was used; see Figure 5C for a version without a limit on zone III oxygen uptake. Nodule biomass was either uncapped (red) or limited to 10% (blue) or 5% (purple) of the overall biomass.

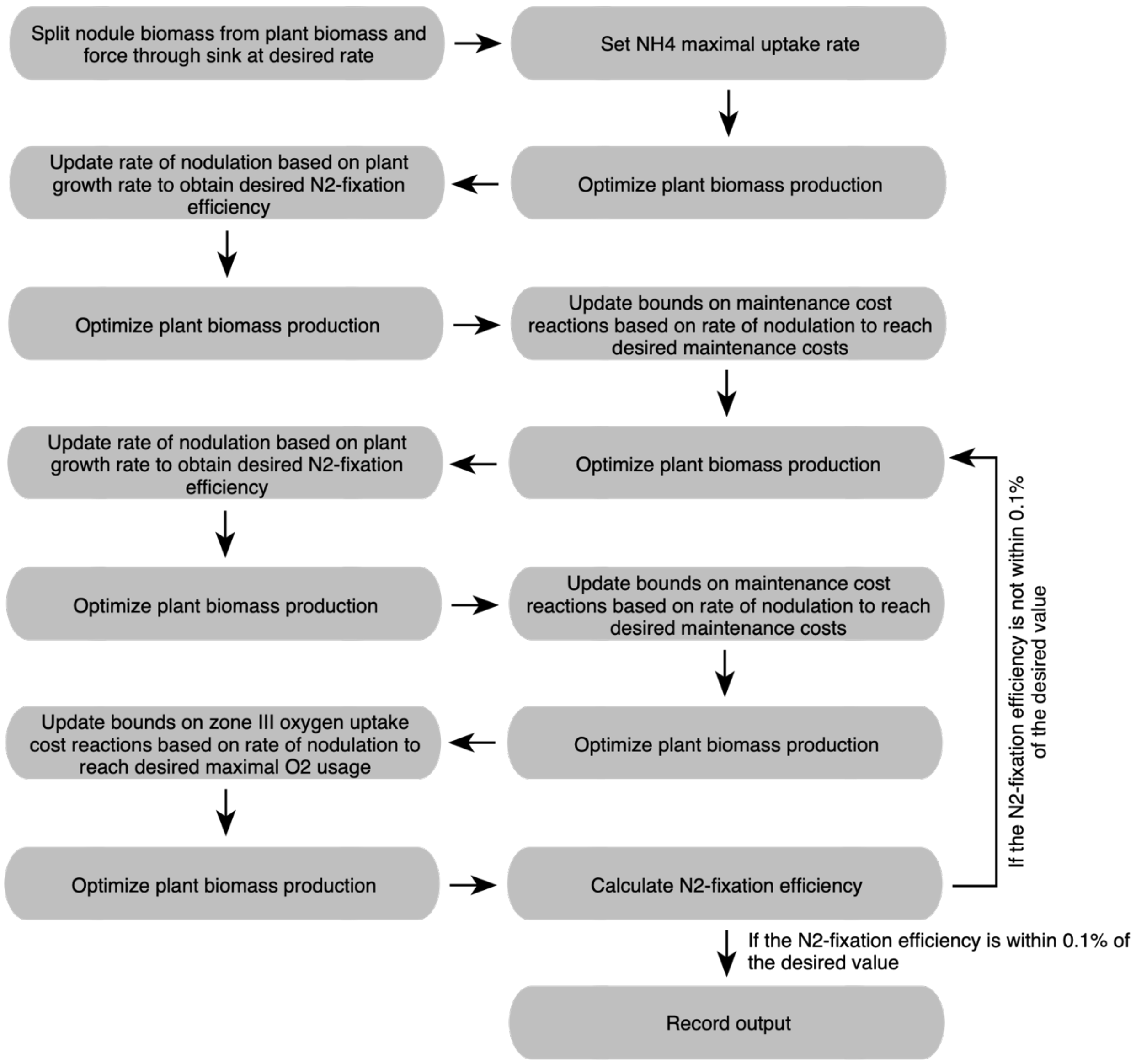

Supplementary Figure 4. A flowchart of an optimization problem. This flow chart outlines the optimization problem for simulations where NH_4 was added to the soil and the rate of nodulation was optimized to maximize plant growth while the N_2-fixation efficiency remained constant. The result of this process is a single datapoint, and the process was repeated each time the availability in exogenous NH_4 was modified.

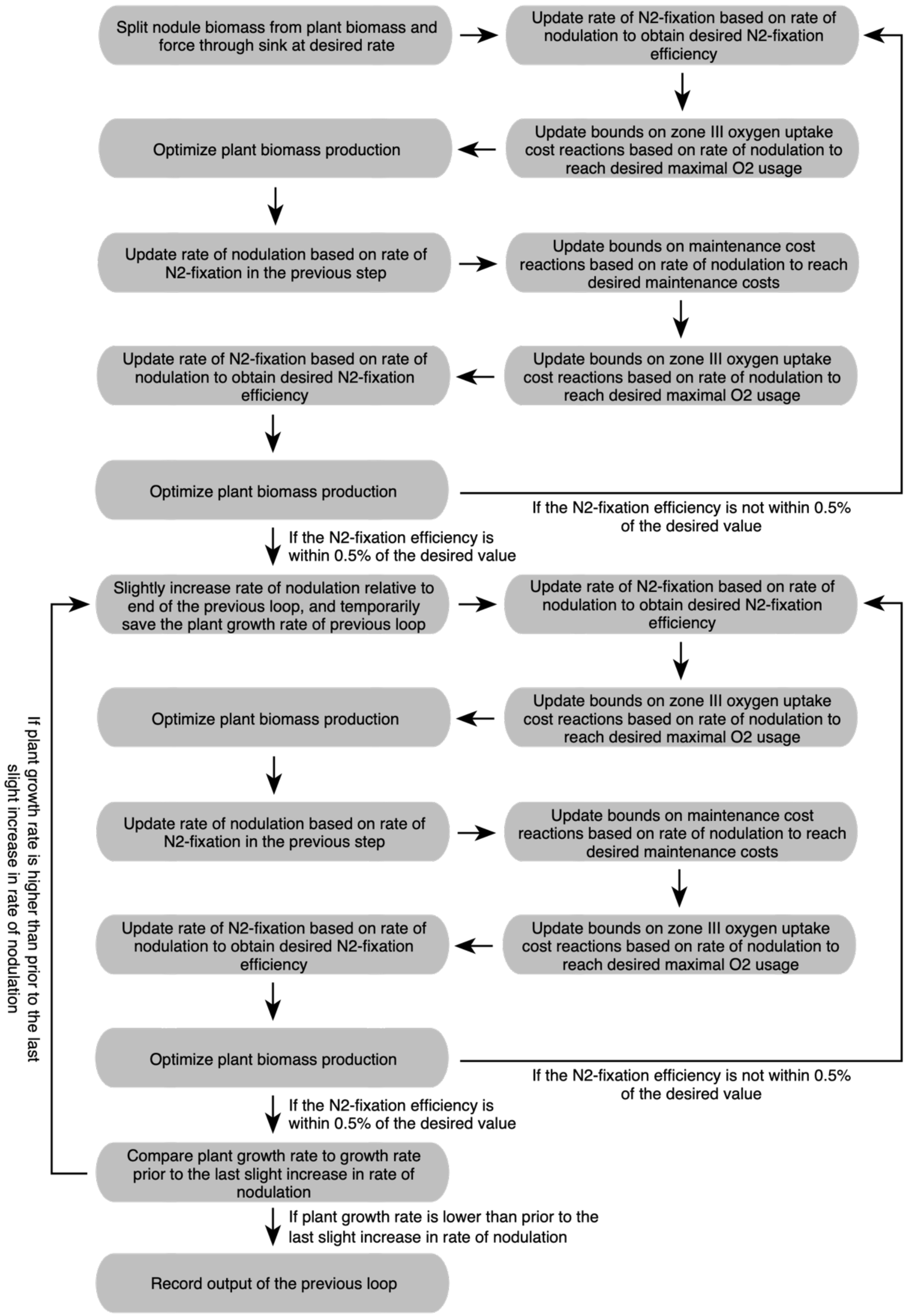

Supplementary Figure 5. A flowchart of an optimization problem. This flow chart outlines the optimization problem for simulations the N_2-fixation efficiency was varied while the rate of nodulation was optimized to maximize plant growth. The result of this process is a single datapoint, and the process was repeated each time the desired N_2-fixation efficiency was modified.

SUPPORTING REFERENCES

1. diCenzo, G. C. *et al.* Robustness encoded across essential and accessory replicons of the ecologically versatile bacterium *Sinorhizobium meliloti*. *PLOS Genet* **14**, e1007357 (2018).

2. Wang, C. *et al.* Roles of poly-3-hydroxybutyrate (PHB) and glycogen in symbiosis of *Sinorhizobium meliloti* with *Medicago* sp. *Microbiology* **153**, 388–398 (2007).

3. Becerra-Rivera, V. A., Bergström, E., Thomas-Oates, J. & Dunn, M. F. Polyamines are required for normal growth in *Sinorhizobium meliloti*. *Microbiology* **164**, 600–613 (2018).

4. diCenzo, G. C. *et al.* Metabolic modelling reveals the specialization of secondary replicons for niche adaptation in *Sinorhizobium meliloti*. *Nat Commun* **7**, 12219 (2016).

5. Kanehisa, M., Sato, Y., Kawashima, M., Furumichi, M. & Tanabe, M. KEGG as a reference resource for gene and protein annotation. *Nucleic Acids Res* **44**, D457–D462 (2016).

6. Caspi, R. *et al.* The MetaCyc database of metabolic pathways and enzymes. *Nucleic Acids Res* **46**, D633–D639 (2018).

7. Henry, C. S. *et al.* High-throughput generation, optimization and analysis of genome-scale metabolic models. *Nat Biotechnol* **28**, 977–982 (2010).

8. Moretti, S. *et al.* MetaNetX/MNXref–reconciliation of metabolites and biochemical reactions to bring together genome-scale metabolic networks. *Nucleic Acids Res* **44**, D523–6 (2016).

9. Kim, S. *et al.* PubChem substance and compound databases. *Nucleic Acids Res* **44**, D1202–13 (2016).

10. Thiele, I. & Palsson, B. Ø. A protocol for generating a high-quality genome-scale metabolic reconstruction. *Nat Protoc* **5**, 93–121 (2010).

11. Feist, A. M. *et al.* A genome-scale metabolic reconstruction for *Escherichia coli* K-12 MG1655 that accounts for 1260 ORFs and thermodynamic information. *Mol Syst Biol* **3**, 121 (2007).

12. Pfau, T. *et al.* The intertwined metabolism during symbiotic nitrogen fixation elucidated by metabolic modelling. *Sci Rep* **8**, 12504 (2018).

13. Young, N. D. *et al.* The *Medicago genome* provides insight into the evolution of rhizobial symbioses. *Nature* **480**, 520–524 (2011).

14. Pecrix, Y. *et al.* Whole-genome landscape of *Medicago truncatula* symbiotic genes. *Nat Plants* **4**, 1017–1025 (2018).

15. Tang, H. *et al.* An improved genome release (version Mt4.0) for the model legume *Medicago truncatula*. *BMC Genomics* **15**, 312 (2014).

16. Krishnakumar, V. *et al.* MTGD: The *Medicago truncatula* genome database. *Plant Cell Physiol* **56**, e1–e1 (2015).

17. Hakoyama, T. *et al.* Host plant genome overcomes the lack of a bacterial gene for symbiotic nitrogen fixation. *Nature* **462**, 514–517 (2009).

18. Overbeek, R. *et al.* The subsystems approach to genome annotation and its use in the project to annotate 1000 genomes. *Nucleic Acids Res* **33**, 5691–5702 (2005).

19. Anderson, M. P., Heichel, G. H. & Vance, C. P. Nonphotosynthetic CO_2 fixation by alfalfa (*Medicago sativa* L.) roots and nodules. *Plant Physiol* **85**, 283–289 (1987).

20. Roux, B. *et al.* An integrated analysis of plant and bacterial gene expression in symbiotic root nodules using laser-capture microdissection coupled to RNA sequencing. *Plant J* **77**, 817–837 (2014).

21. de Mendiburu, F. *agricolae: statistical procedures for agricultural research. R package version 1.2-8.* (2017).

22. Jensen, P. A., Lutz, K. A. & Papin, J. A. TIGER: Toolbox for integrating genome-scale metabolic models, expression data, and transcriptional regulatory networks. *BMC Syst Biol* **5**, 147 (2011).

23. Becker, S. A. & Palsson, B. Ø. Context-specific metabolic networks are consistent with experiments. *PLOS Comput Biol* **4**, e1000082 (2008).

24. diCenzo, G. C., Mengoni, A. & Fondi, M. Tn-Core: a toolbox for integrating Tn-seq gene essentiality data and constraint-based metabolic modelling. *ACS Synth Biol* **8**, 158–169 (2019).

25. Vlassis, N., Pacheco, M. P. & Sauter, T. Fast reconstruction of compact context-specific metabolic network models. *PLOS Comput Biol* **10**, e1003424 (2014).

26. Lieven, C. *et al.* MEMOTE for standardized genome-scale metabolic model testing. *Nat Biotechnol* **38**, 272–276 (2020).

27. Silvester, N. *et al.* The European Nucleotide Archive in 2017. *Nucleic Acids Res* **46**, D36–D40 (2017).

28. Langmead, B. & Salzberg, S. L. Fast gapped-read alignment with Bowtie 2. *Nat Methods* **9**, 357–359 (2012).

29. Li, H. *et al.* The Sequence Alignment/Map format and SAMtools. *Bioinformatics* **25**, 2078–2079 (2009).

30. Anders, S., Pyl, P. T. & Huber, W. HTSeq—a Python framework to work with high-throughput sequencing data. *Bioinformatics* **31**, 166–169 (2015).
