# Supplementary material for: Genome-scale metabolic reconstruction of the symbiosis between a leguminous plant and a nitrogen-fixing bacterium
Source: Nat Commun. 2020 May 22;11:2574. doi: 10.1038/s41467-020-16484-2 (PMC7244743; doi:10.1038/s41467-020-16484-2)
Supplement: Supplementary file 3 — Description of Additional Supplementary Files [file 41467_2020_16484_MOESM3_ESM.pdf]

Description of Additional Supplementary Files

File Name: Supplementary Data 1
Description: SBML, XLS, and MATLAB COBRA formats of the S. meliloti updated metabolic reconstruction (iGD1348) used to generate the ViNE.

File Name: Supplementary Data 2
Description: Comparison of experimental and predicted S. meliloti growth phenotypes.

File Name: Supplementary Data 3
Description: Memote reports for all the models constructed in this work.

File Name: Supplementary Data 4
Description: Overall gene deletion analysis.

File Name: Supplementary Data 5
Description: Nodule zone-specific gene deletion and reaction removal analyses.

File Name: Supplementary Data 6
Description: Bacteroid robustness analysis.

File Name: Supplementary Data 7
Description: The biomass composition of *S. meliloti* as considered in iGD1348.

File Name: Supplementary Data 8
Description: The updated *M. truncatula* metabolic model used in the construction of ViNE.

File Name: Supplementary Data 9
Description: Reactions removed during dead-end removal.

File Name: Supplementary Data 10
Description: The working model used throughout this study (ViNE) and the full integrated model prior to constraining the nodule reaction space based on RNA-seq data (ViNE_precursor).
